# Sociodemographic and Behavioural Factors Affecting the Oral-Health-Related Quality of Life as Measured with the Child-OIDP Index in Adolescents

**DOI:** 10.3290/j.ohpd.b4996999

**Published:** 2024-02-20

**Authors:** Maria Paloma Alvarez-Azaustre, Rossana Greco, Carmen Llena

**Affiliations:** a Associate Professor, Department of Dentistry, Faculty of Biomedical and Health Sciences, Universidad Europea de Valencia, Valencia, Spain. Protocol and study design, acquisition and interpretation of data, wrote and approved the manuscript.; b Dentist in Private Practice, Centro Medico Specialistico Cancellieri, Rome, Italy. Acquisition and interpretation of data, wrote and approved the manuscript.; c Professor, Department of Stomatology, Faculty of Medicine and Dentistry, Universitat de València, Valencia, Spain. Conception and study design, project supervision, proofread and approved the manuscript.

**Keywords:** adolescent, behavioural factors, oral health, quality of life, sociodemographic factors

## Abstract

**Purpose:**

Environmental factors modulate oral-health-related quality of life (OHRQoL). The aim of this study was to analyse sociodemographic and behavioural factors affecting the OHRQoL in Spanish adolescents, by using the Child-OIDP (Child-Oral Impacts on Daily Performances) index.

**Materials and Methods:**

A cross-sectional study was conducted in 337 adolescent schoolchildren aged 13–15 years. A questionnaire on sociodemographic, behavioural and oral self-perception factors was administered with the Child-OIDP questionnaire. Descriptive statistics, Kruskal-Wallis and Mann-Whitney U-tests, as well as a regression model were used in the data analysis.

**Results:**

The overall mean Child-OIDP index was 3.28±6.55. It was statistically significantly higher in females than in males (p < 0.001). Mothers having a managerial job showed statistical association with worse OHRQoL (p < 0.001). Caries experience and history of dental trauma were not associated with the oral-health-related quality of life (p > 0.05). Halitosis statistically significantly affected the activities of daily living (p < 0.001). Perceived dental problems, dental treatment needs, self-assessment of oral health status and satisfaction with oral health were associated with the impact index (p < 0.05).

**Conclusion:**

Mothers who were managers, female sex, presence of halitosis, and perceived dental treatment needs were the most important predictors of the impact index, while dietary habits, oral hygiene, and dental visits did not affect it. Knowledge of these factors will help dental professionals to apply adequate preventive and therapeutic measures.

The impact of oral conditions on people’s lives can be evaluated by Oral Health-Related Quality of Life (OHRQoL) measures which capture the effect of oral health on physical, psychological, functional and social aspects.^42^ OHRQoL is a multidimensional construct characterised by being subjective, and various questionnaires have been developed to measure the impact of oral conditions on quality of life in adults and children.^41^

The use of OHRQoL questionnaires aimed at children and adolescents requires adaptation of the content and number of questions, as well as language and recall period. The domains addressed in those questionnaires may be oral symptoms, functional limitation, emotional and social well-being, and school environment.^11,12,14^ Among these questionnaires, the CPQ11-14 (Child Perceptions Questionnaire 11-14), the COHIP (Child Oral Health Impact Profile) and the Child-OIDP (Child-Oral Impacts on Daily Performances) are the most frequently used instruments.^42^

The Child-OIDP questionnaire is one of the most widely used in dental epidemiology and dental health services research.^42^ It assesses the impact of oral health on eight daily-life activities in children and adolescents, and comprises four domains – oral health, functional, social, and emotional well-being – to capture the consequences of oral diseases on functional and psychosocial aspects.^14^

The perception that a child or adolescent has of the impact of their oral health on activities of daily living is influenced by the environment in which they live, as well as the value system and expectations of their cultural background. Regarding adolescents’ perception of oral health over their life course, three themes have been identified that young people associated with their oral health status: understanding the value of maintaining good oral health for a lifetime, positive association between good oral health and interpersonal relationships, and highlighting the importance of appearance and positive self-image that could be achieved with orthodontic treatment.^21^

The assessment of sociodemographic and behavioural factors affecting the oral-health-related quality of life (OHRQoL) in adolescents enables proposing preventive or therapeutic interventions adapted to the cultural context in which they live, which maximises the effectiveness of oral health care in a limited-resource context. It is also important to study the influence of sociodemographic factors on the OHRQoL, as they may explain why sometimes the relationship between clinical status and OHRQoL is weak or inconsistent, as sociodemographic factors may act as modulators.^17^

The environment where children live has been reported as influencing their health behaviours and their perception of oral health.^20^ Similarly, the model proposed by Sischo and Broder^37^ on factors associated with OHRQoL recognises the effect of sociocultural factors on oral health perception and related quality of life. Several studies have shown socioeconomic inequalities in subjective measures of oral health in adults, finding a clear education gradient, with worse perceptions at each lower level of education;^43^ however, this relationship has been studied less frequently in adolescents.

Among the determinants affecting OHRQoL in adolescents, a variety of sociodemographic factors have been identified including socioeconomic status (SES), place of residence (urban or rural), age, sex, nationality and ethnic background.

Behavioural factors play an important role in the development of oral diseases, e.g., dental caries or gingivitis. The consumption of sugar-sweetened beverages is still common among adolescents in many countries.^38^ Factors such as oral hygiene and dietary habits may contribute to the occurrence of oral diseases and therefore affect the OHRQoL.

In this context, the study conducted by Montero et al^27^ in Spanish schoolchildren aged 6–12 years found that some sociodemographic and behavioural factors modulated the impact of clinical conditions on the quality of life in several domains. Since then, to our knowledge, the influence of these factors on the OHRQoL in Spanish adolescents has not been assessed. Thus, the aim of this study was to analyse sociodemographic and behavioural factors affecting OHRQoL in Spanish adolescents, as measured with the Child-OIDP index. Our hypothesis was that sociodemographic, behavioural, and oral self-perception factors were associated with the the OHRQoL. The null hypothesis was that those factors were not associated with the the OHRQoL.

## Materials and Methods

### Study Design and Sample

A descriptive cross-sectional study was designed and conducted using cluster sampling. Twenty-five schools were selected at random, 13 public and 12 private schools, in the region of Valencia in easter Spain, and invited to participate in the study.

A random sample of 337 adolescents 13–15 years old, in the 2nd and 3rd years of compulsory secondary education, were recruited for the study. School officials, pupils, teachers, and adolescents’ parents were briefed about the purpose and process of the study; written informed consent was sought and signed in advance by the participants and their parents or guardians. Ethical approval was obtained from the Ethics Committee of University of València (ref. H 20190501104101).

The sample size calculation was based on the impact prevalence of oral health on daily activities, as reported in the previous validation study of the Child-OIDP questionnaire for use in adolescents in Spain.^10^ Considering an estimated impact prevalence of between 30% and 40%, an estimated sample size of 323-369 pupils was obtained, using the formula:


$$N=\frac{\text{Z}\alpha^{2}\text{P}(1-\text{P})}{i^{2}}$$

where Zα^2^ = (1.96)^2^ (constant corresponding to a confidence level of 95%); P = expected impact prevalence; and *i*^2^ = 0.05 (error assumed for 5% precision).

### Data Collection

**Fig 1:**
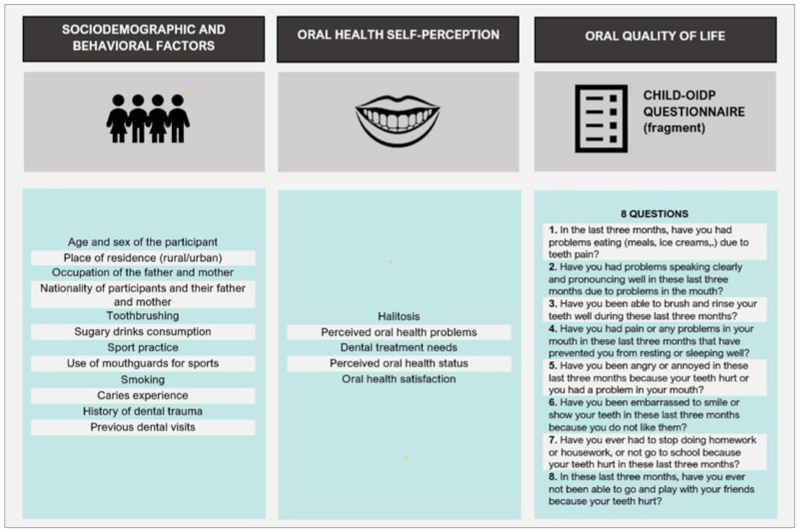
Graphic illustration of data collection procedure.

The fieldwork was carried out by visiting the schools during school hours (Fig 1). An oral clinical exam was performed after administering the questionnaires, the results of which have been published previously.^1^

#### Questionnaire on sociodemographic and behavioural factors

**Fig S1:**
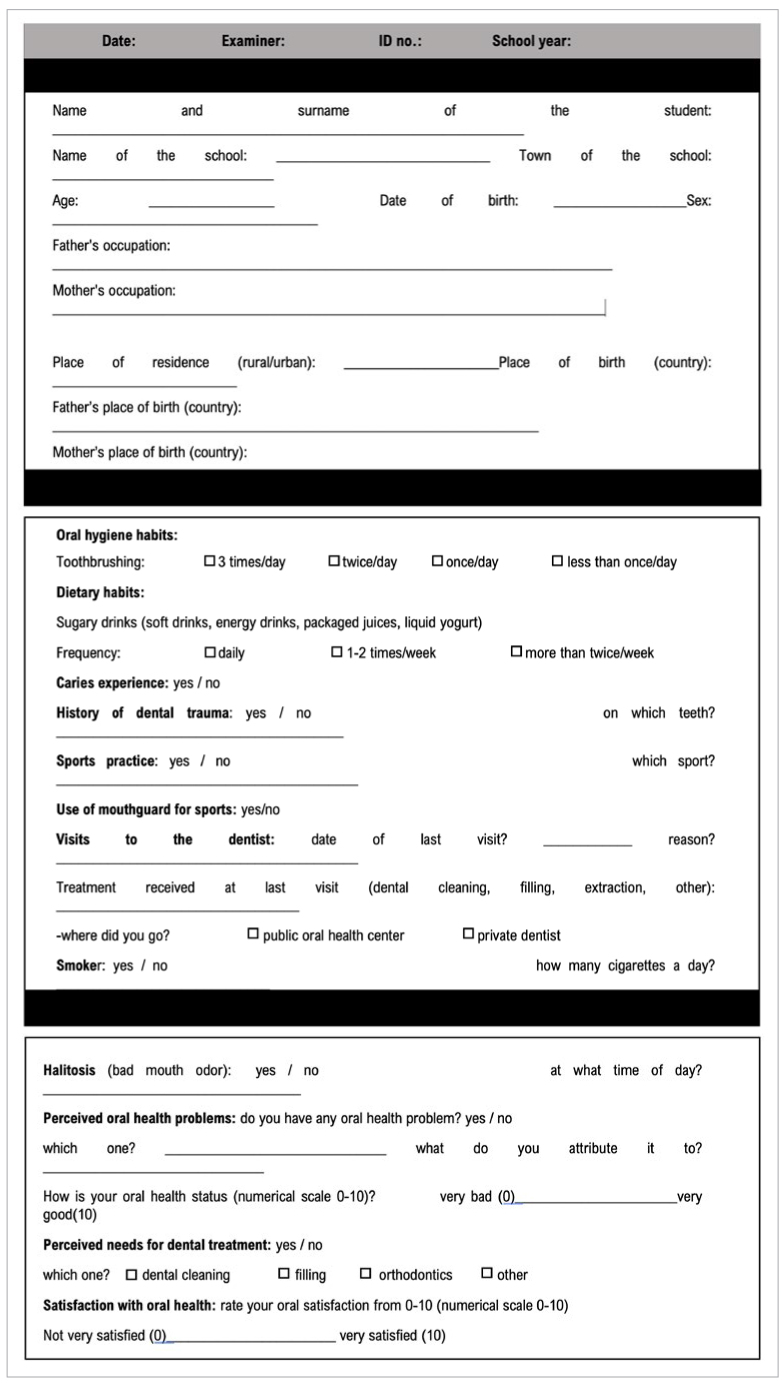
Questionnaire on sociodemographic and behavioural factors.

An interviewer-administered survey was applied to record sociodemographic and behavioural factors, dental visits, and history of dental health (caries, dental trauma). The behavioural variables included daily frequency of toothbrushing and weekly consumption of sugary drinks, as well as sports practice, the use of mouthguard and cigarette consumption, which were scored as yes/no. Caries experience and history of dental trauma and were also scored as yes/no. The assessment of previous dental visits included date of last visit, reason for the visit, treatment received, and the type of clinic attended: either private or public (Fig S1).

#### Questionnaire on oral self-perception

The questionnaire on oral self-perception was also interviewer-administered, and it included several questions regarding perceived dental treatment needs and perceived oral health problems.^27^ Perception of halitosis was scored as yes/no (Fig S1).

#### Questionnaire on the OHRQoL: the Child-OIDP

**Fig S2:**
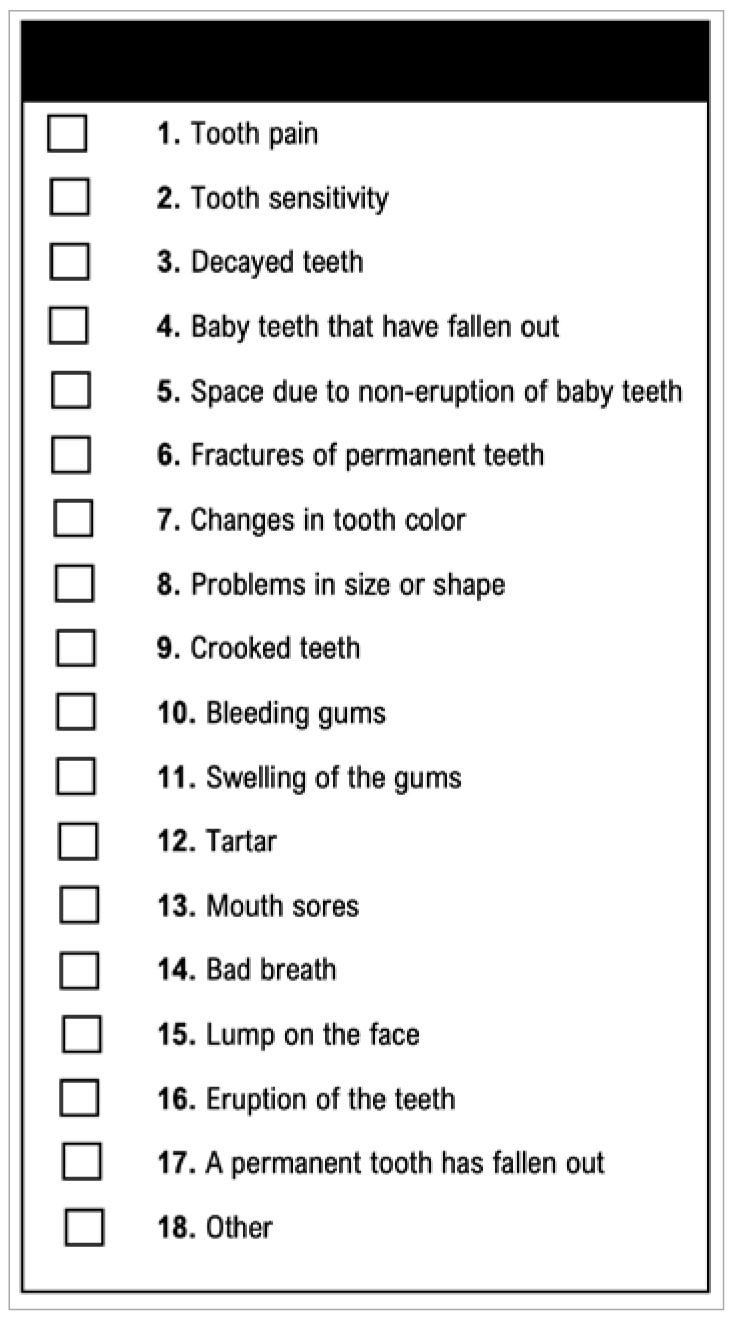
List of 18 oral/dental problems that can be selected as a cause of impact.

Prior to the administration of the Child-OIDP questionnaire, each participant was provided with a list of 18 oral problems, and they were asked to mark with an X those they had experienced in the last three months, in order to subsquently analyse the causes of impacts (Fig S2).

The Child-OIDP questionnaire was self-administered.^13,34^ It includes eight structured questions, where participants were asked about the severity and frequency of the oral impacts on eight activities of daily living performed by the adolescent: (1) eating, (2) speaking, (3) brushing teeth, (4) sleeping, (5) emotional state, (6) smiling, (7) schoolwork and (8) playing. For each question, the severity and frequency of the impact were evaluated on a Likert scale from 0 to 3, with higher values corresponding to poorer status (Fig S3).

**Fig S3:**
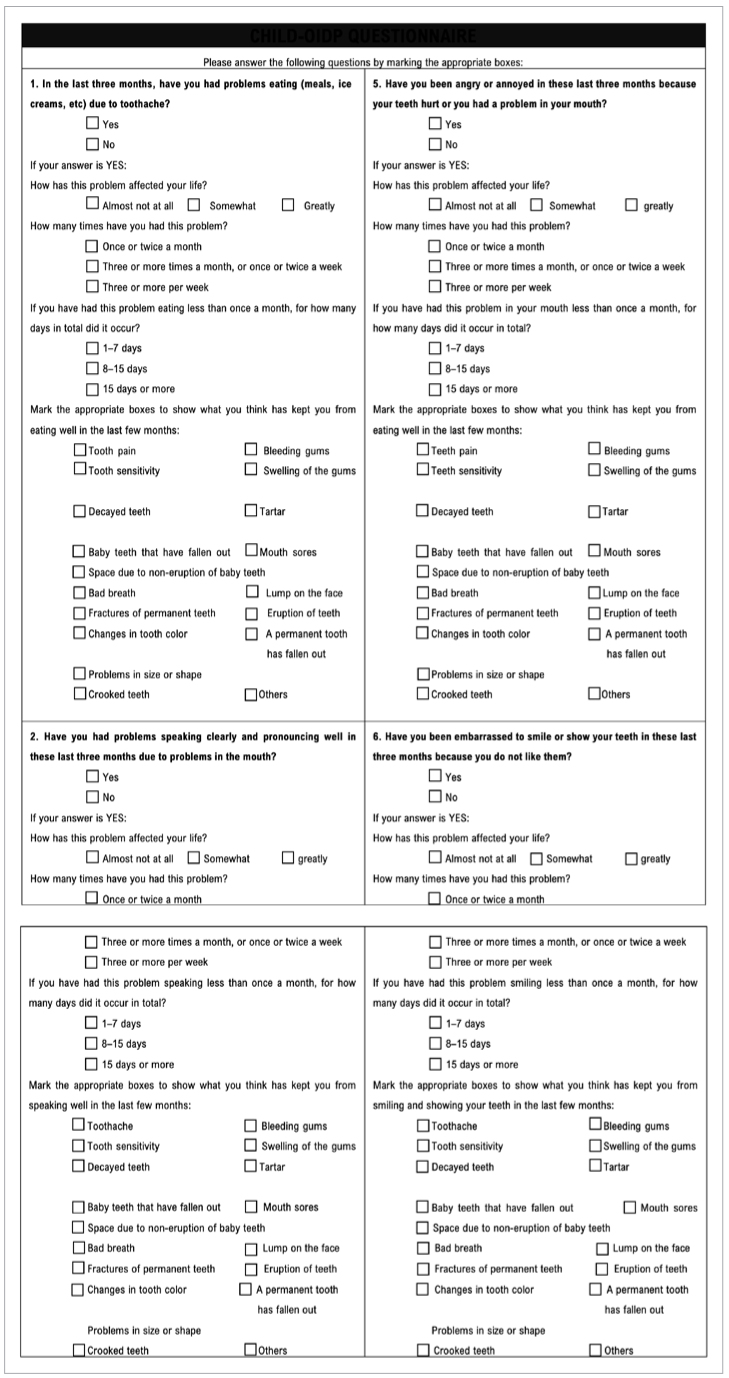
Child-Oral Impacts on Daily Performances questionnaire (Part 1).

**Fig S3:**
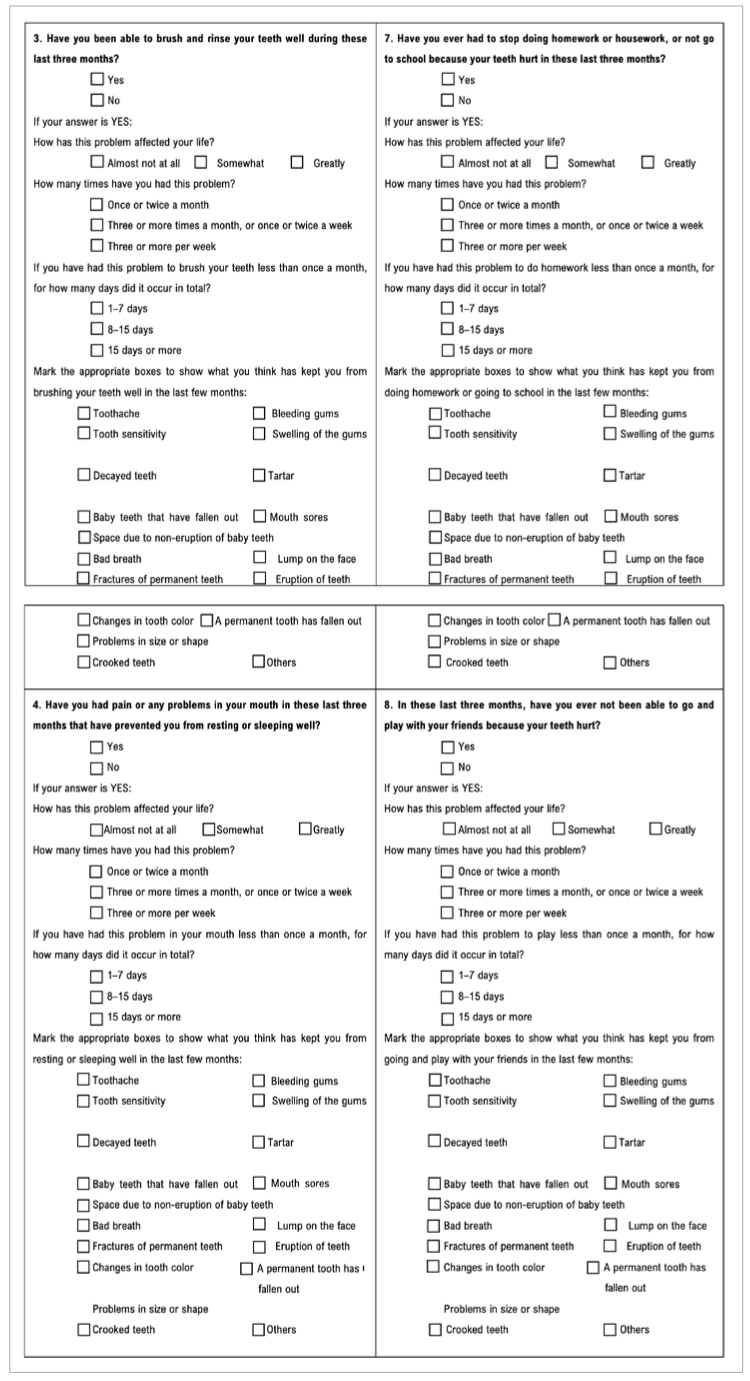
Child-Oral Impacts on Daily Performances questionnaire (Part 2).

To calculate the Child-OIDP index, the severity is multiplied by the frequency of impact on each performance, and the impact intensity on each performance is obtained. The maximum impact intensity per performance is nine. The average mean Child-OIDP index, or impact index, was obtained with the formula:


$$\text{Child-OIDP index}=\frac{\Sigma (\text{severity}\times \text{frequency})}{72}\times 100$$

Higher scores denote greater impact of oral conditions on adolescents’ the OHRQoL, and therefore worse OHRQoL.^10^

### Data Analysis

Descriptive statistics were used to characterise the study population, followed by bivariate analysis and a regression model. Due to the skewed distribution of the Child-OIDP index, non-parametric Kruskal-Wallis and Mann-Whitney U-tests were applied to assess the relationship between sociodemographic and behavioural factors, history of dental disease/trauma, and oral health self-perception variables, as well as the impact index. All analyses were performed using IBM Statistical Package SPSS version 28.0 (Chicago, IL, USA). A significance level of p < 0.05 was used for all analyses.

## Results

A total of 337 schoolchildren 13–15 years old were enrolled in the study, 53.1% were females and 46.9% were males. Psychometric properties of the Child-OIDP questionnaire were fully assessed; a complete description can be found elsewhere.^1^

### Sociodemographic Factors and Their Association with the Child-OIDP Index

In relation to the place of residence of the participants, most of them lived in semiurban areas (86.3%), followed by urban areas (9.5%). Only a small percentage were from rural areas (4.2%).

According to the professional categories established in the Spanish National Occupation Classification (Clasificación Nacional de Ocupaciones, CNO 2011),^15^ fathers mostly performed manual labor (which includes all categories shown in Table 1 except “manager” and “technical”), only 7.7% had academic or managerial jobs. Regarding the occupation of the mothers, 62.7% were housewives or worked in personal services, and 8.6% had academic or managerial jobs. The complete distribution is shown in Table 1.

**Table 1:** Occupation of the parents

Occupation	Father		Mother	
	n	%	n	%
Manager	5	1.5	4	1.2
Technical	21	6.2	25	7.4
Technician	17	5	15	4.5
Clerk/office	25	7.4	51	15.1
Restaurant/personal services	64	19	75	22.3
Farming/livestock worker	4	1.2	2	0.6
Manufacturing/construction worker	50	14.8	11	3.3
Machinery operator	107	31.8	2	0.6
Basic work/housewife	17	5	136	40.4
Army	2	0.6	1	0.3
Not working	19	5.6	14	4.2
Deceased	6	1.8	1	0.3
Total	337	100	337	100

Regarding the nationality of the participants, 94.1% were Spanish. The father and/or mother were foreigners in 16% of the cases. As shown in Table 2, the country of origin of the father, mother or participants did not significantly influence the the OHRQoL of the adolescents. The values for the Child-OIDP index were found to be statistically significantly higher in females than in males (p < 0.001), which means that female sex had a negative impact on the the OHRQoL, and the girls presented a mean impact index twice as high as that of boys.

**Table 2:** Association between sociodemographic variables and the Child-OIDP index

Variables	C-OIDP					
	n	Mean ± SD	95% CI	Median	Interquartile range	p-value
Country of father Spain	317	3.2±6.6	2.5-3.9	0	3.47	0.62
Country of father abroad	20	3.5±5.1	1.1-5.9	0.69	7.2	
Country of mother Spain	283	3.2±6.8	2.4-4	0	2.7	0.44
Country of mother abroad	54	3.3±4.9	1.9-4.6	1.3	5.5	
Country of participant Spain	317	3.2±6.6	3.5-5.1	0	3.4	0.62
Country of participant abroad	20	3.5±5.1	1.1-5.9	0.6	7.2	
Male	158	2.1±4.2	1.4-2.7	0	2.7	<0.001
Female	179	4.2±7.9	3.1-5.4	1.3	5.5	
Father’s job manager-clerk	68	4.3±9.9	1.9-6.8	0.3	3.8	0.05
Father’s job personal services-basic	269	2.9±5.3	2.3-3.6	0	4.1	
Mother’s job manager-clerk	95	4±7.5	2.4-5.5	1.3	5.5	<0.001
Mother’s job personal services-basic	242	2.9±6	2.2-3.7	0	2.7	

The father’s and mother’s occupations were grouped into two categories (manager-clerk/personal services-basic). In the cases where the mother had a managerial job, the Child-OIDP values were significantly higher (p < 0.001), meaning that the managerial job of the mother had a negative impact on the the OHRQoL of her children, whereas for the father’s managerial jobs, the values were below statistical significance (p=0.05).

### Behavioural Factors and Their Association with the Child-OIDP Index

Participants were asked about the frequency of toothbrushing, and around 75% of the sample reported brushing their teeth twice or more times a day. Toothbrushing did not show a statistically significant association with the Child-OIDP index (p=0.05) (Table 3). Therefore, toothbrushing did not affect the OHRQoL.

**Table 3:** Association between behavioural variables and the Child-OIDP index

Variables	C-OIDP					
	n	Mean ± SD	95% CI	Median	Interquartile range	p value
Toothbrushing ≥ 2 times/day	251	3.57±6.88	2.71–4.43	1.38	4.17	0.05
Toothbrushing < twice/day	86	2.42±5.42	1.25–3.58	0	2.78	
Sugary drinks 1–2 times/week	178	3.1±7	2–4.1	0	2.7	0.76
Sugary drinks > twice/week	50	4.1±7	2.1–6.1	0	7.2	
Non–smoker	330	3.2±6.4	2.4–3.9	0	2.7	0.09
Smoker	7	6.9±8.8	1.2–15.1	4.1	11.1	
No sports	101	4.2±9.2	2.4–6	0	4.1	0.79
Sports	236	2.8±4.9	2.2–3.4	0	2.7	
No mouthguard	309	3.3±6.7	2.5–4	0	4.1	0.95
Mouthguard	28	2.9±4.7	1.1–4.8	0	3.8	
Last dental visit > 1 year	86	3.1±5.5	1.9–4.3	0	3.1	0.64
Last dental visit < 1 year	251	3.3±6.8	2.4–4.1	0	4.1	

When asked about the frequency of consumption of sugary drinks, 27% of the adolescents reported drinking sugary liquids more than twice a week or daily. The frequency of consumption of sugary drinks showed no significant association with the Child-OIDP index (p > 0.05). Thus, sugary drinks did not affect the OHRQoL.

Regarding sports practice, 70% of the adolescents practiced sports on a regular basis and only 8.3% used mouthguards for sports. No statistically significant association was found between these variables and the Child-OIDP index (p > 0.05), meaning that sports practice and use of mouthguards likewise did not affect the OHRQoL.

When adolescents were asked about their smoking habits, 97.9% stated that they did not smoke. Although no statistically significant association was found between smoking and the Child-OIDP index (p=0.09), it could be seen that in the small number of participants who reported cigarette consumption, the value of the index was twice as high as that of non-smokers. This means that tobacco smoking had a negative effect on the the OHRQoL in adolescents.

A total of 75.4% of the participants had visited the dentist in the last 12 months, and oral check-up was the main reason for the visit, followed by the presence of discomfort. The most frequent treatment performed at the last visit was dental cleaning and application of fluoride gel on the teeth, followed by a dental check-up. Approximately one-third of the adolescents received other treatment, such as filling, dental extraction, or orthodontic examination. No statistically significant association was found between visits to the dentist and the Child-OIDP index (p > 0.05) (Table 3), meaning that previous dental visits did not affect the OHRQoL.

The participants were also asked if they presented any experience of caries, and 56.6% of them answered affirmatively. History of dental trauma was reported by 25.5%. Caries experience or history of dental trauma were not statistically significantly associated with the Child-OIDP index. Therefore, oral-health history did not affect the OHRQoL.

### Oral Health Perception Factors and Their Association with the Child-OIDP Index

The presence of halitosis was reported by 34.7% of the participants. Halitosis showed a statistically significant association with the Child-OIDP index (p < 0.001) (Table 4), meaning that halitosis had a negative impact on the OHRQoL. Most participants reported having no oral health problems (68%). In the participants with problems, aesthetics was the main perceived problem (9.5%), followed by gingival bleeding (4.2%) and pain (3.6%). We found that 46.3% of participants mentioned dental treatment needs. The most frequently perceived treatment need was orthodontics (25.2%), followed by dental cleaning (12.2%) and fillings (6.5%). The majority of adolescents reported a good oral health status as well as a high satisfaction with their oral health, based on their self-assessment on the 0-10 scale (Fig S1) (Table 4). All the oral-health perception variables analysed in the study showed a significant association with the Child-OIDP index, meaning that the self-perception of the adolescent was related to the the OHRQoL captured by the questionnaire.

**Table 4:** Association between oral health perception variables and the Child-OIDP index

Variables	C-OIDP					
	n	Mean ± SD	95% CI	Median	Interquartile range	p-value
No oral health problems	228	2.1±4.7	1.5–2.8	0	2.7	<0.001
Oral health problems	109	5.5±8.8	3.8–7.2	1.3	8.3	
No treatment needs	181	2.3±6.5	1.3–3.3	0	2.7	<0.001
Treatment needs	156	4.3±6.3	3.3–5.3	1.3	5.5	
Oral health status < 5	29	4.7±6.2	2.4–7.1	2.7	5.5	0.01
Oral health status > 5	308	3.1±6.5	2.4–3.8	0	2.7	
Oral health satisfaction < 5	28	4.7±6.1	2.3–7	2	5.5	0.03
Oral health satisfaction > 5	309	3.1±6.5	2.4–3.8	0	2.7	
No halitosis	220	2.2±5.1	1.5–2.9	0	2.7	<0.001
Halitosis	117	5.1±8.3	3.6–6.7	2.7	7.6	

### Oral Impacts on Daily Activities Captured by the Child-OIDP Questionnaire

The impacts of oral health on the daily activities are shown in the Table 5. It describes the eight performances or dimensions analysed in the questionnaire, the impact prevalence by dimension (percentage of participants with each dimension affected), the mean Child-OIDP score or impact index corresponding to each performance, the impact intensity and extent of impacts. The extent of impacts refers to the number of affected dimensions perceived by the adolescents; this is also known as Performances With Impact (PWI).

**Table 5:** Oral impacts prevalence, Child-OIDP score, intensity and extent of impacts

	Total	Eating	Speaking	Brushing	Sleeping	Emotion	Smiling	Schoolwork	Playing
Impact prevalence n (%)	337 (48.1)	72 (21.4)	20 (5.9)	24 (7.1)	31 (9.2)	61 (18.1)	65 (19.3)	5 (1.5)	3 (0.9)
Mean C-OIDP score	3.28± 6.55	0.47±1.16	0.16± 0.82	0.25± 1.14	0.27±1.13	0.51±1.46	0.63±1.67	0.03±0.37	0.01±0.18
Impact intensity (%)									
Low (1–2)	54.6	15.7	3.6	4.8	5.4	12.4	10.9	0.9	0.9
Medium (3–4)	13.2	3.3	1.5	0	2.1	3	3	0.3	0
High (6–9)	15	2.1	0.9	2.1	1.5	2.7	5.4	0.3	0
Extent of impacts (PWI)									
Affected dimensions	0	1	2	3	4	5	6	7	8
n (%)	175 (51.9)	91 (27)	41 (12.2)	19 (5.6)	6 (1.8)	3 (0.9)	0	2 (0.6)	0

PWI = performances with impacts.

The impact prevalence (percentage of participants who reported at least one affected dimension) was moderate (48.1%), and eating was the activity that was most affected (21.4%). The impact intensity was generally mild (54.6%), and it was high on smiling (5.4%) and emotional state (2.7%). The extent of impact was low with only one dimension affected in 27% of the adolescents. The mean Child-OIDP index or impact index was low (3.28± 6.55), showing a good the OHRQoL in this age group.

### Logistic Regression Analysis

The factors that showed a statistically significant association with the Child-OIDP index in the bivariate analysis were recoded into two categories and entered in a logistic regression model to assess its independent effect on the impact index. The factors that in the logistic regression analysis were statistically significantly related to the OHRQoL in adolescents were: mothers having a managerial job (OR=2.92), the presence of halitosis (OR=2.29), the perceived dental treatment needs (OR=2.17), and female sex OR=1.67 (Table 6), meaning that those factors had a negative impact on the the OHRQoL.

**Table 6:** Logistic regression analysis

	B	Sig.	Exp (B)	95% CI for EXP (B)	
				Lower	Upper
Mother’s job (manager-clerk/other)	-1.07	<0.01	2.92	1.71	4.98
DMFT = 0/>0	0.33	.20	1.40	0.83	2.36
Molar relationship (Class I/Others)	0.35	.17	1.42	0.85	2.37
Sex (male/female)	0.51	.03	1.67	1.03	2.72
Halitosis (no/yes)	0.82	<0.01	2.29	1.37	3.82
Perceived oral health status >5/0-5	-0.84	.12	0.42	0.14	1.25
Oral health satisfaction >5/0-5	0.21	.69	1.24	0.42	3.68
Perceived dental treatment needs (no/yes)	0.77	<0.01	2.17	1.29	3.63
Perceived oral health problems (no/yes)	0.39	.16	1.49	0.85	2.60
Constant	-0.56	.61	0.56		

DMFT: Decayed, Missing, Filled Teeth.

## Discussion

Since Chen et al^9^ proposed their conceptual model on the interrelationship of socioeconomic status, oral health behaviour, and oral health status with OHRQoL, there has been a growing interest in this field. Among the the OHRQoL questionnaires for children and adolescents, the CPQ 11-14 is the most frequently used, along with COHIP.^42^ Both have several versions, long and short, depending on the number of questions, and they assess the frequency of oral impacts on daily activities.

The Child-OIDP questionnaire has been cross-culturally adapted and validated in multiple contexts, reporting adequate psychometric properties. It assesses the severity and frequency of oral impacts, captures the adolescent’s perspective in a more complete way, and allows relating the impacts to the causal pathologies. For these reasons, it was chosen for this study. Likewise, OHRQoL in children and adolescents in Spain has been mainly studied using the Child-OIDP questionnaire, since it was validated by Cortés-Martinicorena et al^10^ in 2010.

### Sociodemographic Factors and The OHRQoL

In our study we found that girls have worse OHRQoL than boys, with higher impacts in all the dimensions evaluated by the Child-OIDP questionnaire, except for speaking. This agrees with previous findings,^3,8^ although the results of some other studies do not.^4,19^ Pavithran et al^31^ reported higher impact in females only in the group of orphans (as opposed to non-orphans). Regarding the different dimensions affected in females women and males, Paredes-Martínez et al^30^ found that the dimension most frequently affected in females was smiling, while in males it was eating. Also Sun et al,^39^ using the CPQ11-14 questionnaire, found a greater impact on the dimension “oral symptoms” in men and on the dimension “emotional state” in women.

Because our study was limited to adolescents 13–15 years old, it was not feasible to analyse the association between the child’s age and the OHRQoL. Other reports have shown the influence of age on the impact of oral health on daily activities.^3,13,31^

Several systematic reviews have substantiated the relationship between socioeconomic status (SES) and the OHRQoL. Knorst et al^16^ analysed the influence of socioeconomic level on the OHRQoL, and reported the presence of a socioeconomic gradient: the lower the socioeconomic level, the worse the the OHRQoL in all age groups, in countries of all economic categories. For instance, Malele-Kolisa et al^22^ found an association of socioeconomic status with the OHRQoL in children in Africa.

Moghaddam et al^26^ reported that lower income level and lower educational level of the mother were associated with worse the OHRQoL in children. Also Sun et al^40^ and Amalia et al^3^ found that higher educational level of the mother was associated with better the OHRQoL of her children. Piovesan et al^32^ using the CPQ11-14 found worse the OHRQoL was reported by children whose mothers did not complete primary education. Alves et al^2^ found that a low educational level of the head of the household was associated with a worse the OHRQoL of their children. Those findings concur with those of Kumar et al,^19^ who reported an association between low socioeconomic status and poorer the OHRQoL.^19^ Also, Kragt et al^17^ found a consistent association between a low family SES and lower OHRQoL.

In contrast, our study showed that adolescents whose parents had managerial jobs had a higher impact index, and therefore worse OHRQoL, and that mothers having a managerial-academic job was statistically significantly associated with the impact index. This could be due to a higher awareness and motivation towards oral health problems from adolescents in a middle or high SES environment, which is captured by the Child-OIDP questionnaire in the form of higher impacts. Our results coincide with Berhan Nordin et al,^5^ who found that a higher maternal education level was associated with a higher impact index in their children.^5^

Regarding the influence of the place of residence, in schoolchildren from a marginal urban environment in Peru, Marcelo-Ingunza et al^23^ found a maximum impact prevalence (100%). Similar results were obtained by Reinoso-Vintimilla et al^33^ in Ecuador with an impact prevalence of 98% in rural areas. Other authors, e.g., Simangwa et al^36^ in Tanzania, found the opposite result, with a low impact prevalence in rural areas that they attribute to the traditional Maasai way of life. In contrast, Amalia et al^3^ in Indonesia found an association between rural setting and higher Child-OIDP index.

In our study, conducted mainly in urban and semi-urban settings, we found an impact prevalence of 48.1%. Higher impact prevalence has been reported in other urban populations, as in the study by Vélez-Vásquez et al^44^ (88.1%) and Castro et al^8^ (88.7%). We may conclude that the influence of a rural or urban environment on the the OHRQoL of adolescents is not conclusive, as it is modulated by the lifestyle and cultural context, as much as by SES.

### Behavioural Factors and The OHRQoL

In our study, none of the behavioural factors analysed showed an association with the impact index. We found no statistically significant association between toothbrushing frequency, consumption of sugary drinks, and the impact of oral health on the quality of life. Neither the practice of sports nor the use of mouthguards for sports were statistically significantly associated with the impact index, as was also the case for caries experience and history of dental trauma. The participants were asked about smoking as a behavioural factor, which showed a statistically significant negative impact on oral health. In fact, the average age to start smoking in Spain is 13 years old, with 18.4% of adolescents admitting to smoking at the age of 14. It was therefore considered important to ask this question. Although the percentage of those who reported smoking in our study was low, its impact on the OHRQoL was high, with an impact index value twice that of those who reported not smoking. This means that smoking had a negative impact on the OHRQoL. Although smoking was very seldom reported in this age group, those who reported it had higher impact indexes.

The role of oral hygiene habits on OHRQoL has been highlighted in other studies which reported that a decreased frequency of toothbrushing was associated with worse the OHRQoL.^5,24,28,36^ Also, halitosis in children is widely associated with poor oral hygiene and could affect their the OHRQoL.^35^ Bianco et al^6^ found an association between frequent mouthwash use and worse the OHRQoL, while Kumar et al^19^ reported that the use of tobacco ash for toothbrushing was associated with more impacts on the OHRQoL.

Eating sugary snacks between meals has been associated with a worse OHRQoL.^24,25,29^ Bianco et al^6^ found an association between low fruit intake and increased Child-OIDP index. Berhan Nordin et al^5^ reported a worse the OHRQoL in children who chewed betel nut frequently.

The frequency of visits to the dentist, the reason for the visits, the place where they were made or the treatment performed showed no statistically significant association with the the OHRQoL in our study; however, Kumar et al^19^ found an association between higher number of visits to the dentist and better the OHRQoL. More research is needed on the influence of behavioural factors on the OHRQoL in adolescents.

With respect to other environmental factors, the role of family background and the contribution of behavioural patterns to perceived oral health outcomes in adolescents has been previously highlighted by other authors.^3,31^

### Oral Health Perception Factors and The OHRQoL

In our study, the perception of halitosis showed a statistically significant association with the impact index, corroborating Castro et al.^8^ All the oral health perception variables analysed showed statistically significant association with the Child-OIDP index, which agreed with the results of several other authors.^7,45^ We found that 46.3% of participants reported having dental treatment needs, while Krisdapong et al^18^ reported a higher number of participants with perceived dental treatment needs.

One limitation of this study is that the cross-sectional design prevents a hypothesis of causality between the explanatory and outcome variables. Also, the age range of the participants had to be limited to 13–15 years. The inclusion of a wider range throughout adolescence would have provided a more complete picture of this population group. Moreover, the occupation of the mother and father were used as proxies to assess the socioeconomic status, but they may not be stable indicators, as they may change over time.

## Conclusions

Various environmental factors modulate the impact of oral health on daily activities as perceived by children and adolescents, including the lifestyle and cultural context, as much as the socioeconomic level. Mothers having a managerial job, the presence of halitosis, the perceived dental treatment needs, and female sex were the most important predictors of the impact index on the the OHRQoL in Spanish adolescents, meaning that they had a negative impact on the the OHRQoL. Knowledge of the sociodemographic and behavioural factors affecting the OHRQoL will help dental professionals to apply preventive and therapeutic measures appropriate to the oral health needs of the adolescents.
